# Prognostic Implication of *CYP2C19* Genotype During Chronic Clopidogrel Monotherapy After Percutaneous Coronary Intervention

**DOI:** 10.1016/j.jacasi.2026.08.034

**Published:** 2026-11-03

**Authors:** Jinhwan Jo, Soo-Youn Lee, Ki Hong Choi, Young Bin Song, Byeong-Keuk Kim, Hyung Joon Joo, Kiyuk Chang, Jin-Yong Hwang, Sung Gyun Ahn, Jung-Won Suh, Sang Yeub Lee, Jeehoon Kang, Eun-Seok Shin, Yong Hwan Park, Jong-Young Lee, Jin-Ok Jeong, Chan Joon Kim, Kyeong Ho Yun, Han Cheol Lee, Mahn-Won Park, Jang-Whan Bae, Joon-Hyung Doh, Young-Hoon Jeong, Joo-Yong Hahn

**Affiliations:** a Heart and Brain Hospital, Chung-Ang University Medical Center, Gwangmyeong and Department of Internal Medicine, Chung-Ang University College of Medicine, Seoul, South Korea; b Samsung Medical Center, Sungkyunkwan University School of Medicine, Seoul, South Korea; c Severance Cardiovascular Hospital, Yonsei University College of Medicine, Seoul, South Korea; d Department of Cardiology, Cardiovascular Center, Korea University Anam Hospital, Korea University College of Medicine, Seoul, South Korea; e Division of Cardiology, Department of Internal Medicine, Catholic University of Korea, Seoul, South Korea; f Cardiovascular Center, Gyeongsang National University Hospital, Gyeongsang National University School of Medicine, Jinju, South Korea; g Department of Cardiology, Yonsei University Wonju Severance Christian Hospital, Wonju, South Korea; h Department of Internal Medicine, Seoul National University College of Medicine, Seoul National University Bundang Hospital, Seongnam, South Korea; i Department of Internal Medicine and Cardiovascular Center, Seoul National University Hospital, Seoul, South Korea; j Department of Cardiology, Ulsan University Hospital, University of Ulsan College of Medicine, Ulsan, South Korea; k Samsung Changwon Hospital, Sungkyunkwan University School of Medicine, Changwon, South Korea; l Hallym University Sacred Heart Hospital, Anyang, South Korea; m Chungnam National University Hospital, Chungnam National University College of Medicine, Daejeon, South Korea; n Uijeongbu St Mary's Hospital, The Catholic University of Korea, Seoul, South Korea; o Wonkwang University Hospital, Iksan, South Korea; p Pusan National University Hospital, Busan, South Korea; q Daejeon St Mary's Hospital, The Catholic University of Korea, Daejeon, South Korea; r Good Samsun Hospital, Busan, South Korea; s Inje University Ilsan Paik Hospital, Goyang, South Korea; t CAU Thrombosis and Biomarker Center, Chung-Ang University Gwangmyeong Hospital, Gwangmyeong and Department of Internal Medicine, Chung-Ang University College of Medicine, Seoul, South Korea

**Keywords:** clopidogrel, *CYP2C19* genotype, major adverse cardiac and cerebrovascular events, major bleeding, percutaneous coronary intervention

## Abstract

**Background:**

Recent clinical trials have demonstrated that clopidogrel monotherapy is superior to aspirin for long-term maintenance after percutaneous coronary intervention (PCI). However, evidence remains limited regarding whether the clinical benefit of chronic clopidogrel monotherapy differs according to *CYP2C19* genotype.

**Objectives:**

The study sought to investigate the prognostic impact of *CYP2C19* genotype in patients with clopidogrel monotherapy for long-term maintenance following PCI.

**Methods:**

From the SMART-CHOICE 3 (SMart Angioplasty Research Team: CHoice of Optimal Anti-Thrombotic Strategy in Patients Undergoing Implantation of Coronary Drug-Eluting Stents 3) trial and the nationwide PTRG-DES (Platelet Function and genoType-Related Long-term progGosis in DES-treated Patients) consortium, we analyzed patients with *CYP2C19* genotyping who completed the standard duration of dual antiplatelet therapy after PCI without cardiovascular events. Patients were classified according to *CYP2C19* loss-of-function allele status: rapid or normal metabolizer vs intermediate or poor metabolizer (IM/PM). The primary outcome was a composite of all-cause death, myocardial infarction, and stroke at 3 years after switching to clopidogrel monotherapy.

**Results:**

Of 1,352 patients, 807 patients were carriers of a loss-of-function allele (IM/PM). The risk of primary outcome did not differ between the IM/PM and rapid or normal metabolizer groups (1.2% vs 2.2%; adjusted HR: 0.53; 95% CI: 0.20-1.44; *P* = 0.215). Likewise, the risk of major bleeding was comparable between the groups (1.1% vs 2.0%; adjusted HR: 0.40; 95% CI: 0.14-1.14; *P* = 0.086). Subgroup analyses consistently showed no significant interaction between *CYP2C19* genotype and primary outcome.

**Conclusions:**

In patients who completed the standard duration of dual antiplatelet therapy following PCI, clinical effects of clopidogrel monotherapy were consistent across *CYP2C19* genotypes. These findings provide clinical reassurance that clopidogrel monotherapy can be used effectively for long-term maintenance, regardless of *CYP2C19* genotype. (SMart Angioplasty Research Team: CHoice of Optimal Anti-Thrombotic Strategy in Patients Undergoing Implantation of Coronary Drug-Eluting Stents 3 [SMART-CHOICE 3]; NCT04418479; Platelet Function and genoType-Related Long-term progGosis in DES-treated Patients [PTRG-DES]; NCT04734028)

Following percutaneous coronary intervention (PCI), lifelong single antiplatelet therapy after completion of the standard duration of dual antiplatelet therapy (DAPT) is recommended for secondary prevention.^1^^,^^2^ Although aspirin has long been the standard of care for chronic antiplatelet therapy,^1^^,^^2^ recent landmark clinical trials including the HOST-EXAM (Harmonizing Optimal Strategy for Treatment of Coronary Artery Stenosis–Extended Antiplatelet Monotherapy) and SMART-CHOICE 3 (SMart Angioplasty Research Team: CHoice of Optimal Anti-Thrombotic Strategy in Patients Undergoing Implantation of Coronary Drug-Eluting Stents 3) trials along with large-scale meta-analyses, have demonstrated that clopidogrel monotherapy is superior to aspirin for secondary prevention, with fewer ischemic events.^3^, ^4^, ^5^

Despite the established clinical benefits of clopidogrel, an important concern remains regarding the relationship between clopidogrel's pharmacodynamic effect and *cytochrome P450* (*CYP*) *2C19* enzymatic activity. Previous studies have shown that carriage of *CYP2C19* loss-of-function alleles is associated with reduced levels of clopidogrel's active metabolite and may increase the risk of adverse cardiovascular events during DAPT following PCI.^6^, ^7^, ^8^ In the nationwide, multicenter PTRG-DES (Platelet Function and genoType-Related Long-term progGosis in DES-treated Patients) consortium (n = 8,163), we reported that carriage of *CYP2C19* loss-of-function alleles was associated with a 1.42-fold higher risk of ischemic events during clopidogrel-based DAPT after drug-eluting stent (DES) implantation.^9^ However, most evidence linking the *CYP2C19* genotype to ischemic events has been derived from the early, high-risk phase during DAPT, such as within the first year after presentation with acute coronary syndrome (ACS). Consequently, evidence remains limited regarding the prognostic impact of the *CYP2C19* genotype during the maintenance phase with clopidogrel monotherapy.

Therefore, we investigated the prognostic impact of the *CYP2C19* genotype on clinical outcomes in stable patients who completed the standard duration of DAPT without cardiovascular events and subsequently transitioned to clopidogrel monotherapy, using pooled data from the SMART-CHOICE 3 trial and the PTRG-DES consortium.^4^^,^^10^

## Methods

### Study design and population

This study was an individual patient-level pooled analysis using the SMART-CHOICE 3 (NCT04418479) and PTRG-DES (NCT04734028) dataset. The SMART-CHOICE 3 trial was a prospective, multicenter randomized controlled trial comparing clopidogrel monotherapy with aspirin monotherapy in patients at high risk of recurrent ischemic events who completed the standard duration of DAPT following PCI.^4^ The PTRG-DES consortium was a nationwide, multicenter registry from 32 Korean academic centers, designed to evaluate the relationship between *CYP2C19* genotyping and subsequent clinical outcomes in patients with coronary artery disease undergoing DES implantation.^10^

From the combined data set of these 2 cohorts, we selected patients who: 1) successfully completed the standard duration of DAPT after PCI (12 months for myocardial infarction; 6 months for any other events requiring PCI) without any cardiovascular events; 2) transited to clopidogrel monotherapy (75 mg once a day) as a chronic maintenance strategy; and 3) had available *CYP2C19* genotyping. As a result, 1,352 patients were included and classified into 2 groups according to carriage of *CYP2C19* loss-of-function allele(s): 1) rapid metabolizer (RM) or normal metabolizer (NM); and 2) intermediate metabolizer (IM) or poor metabolizer (PM) (Figure 1).

**Figure 1:**
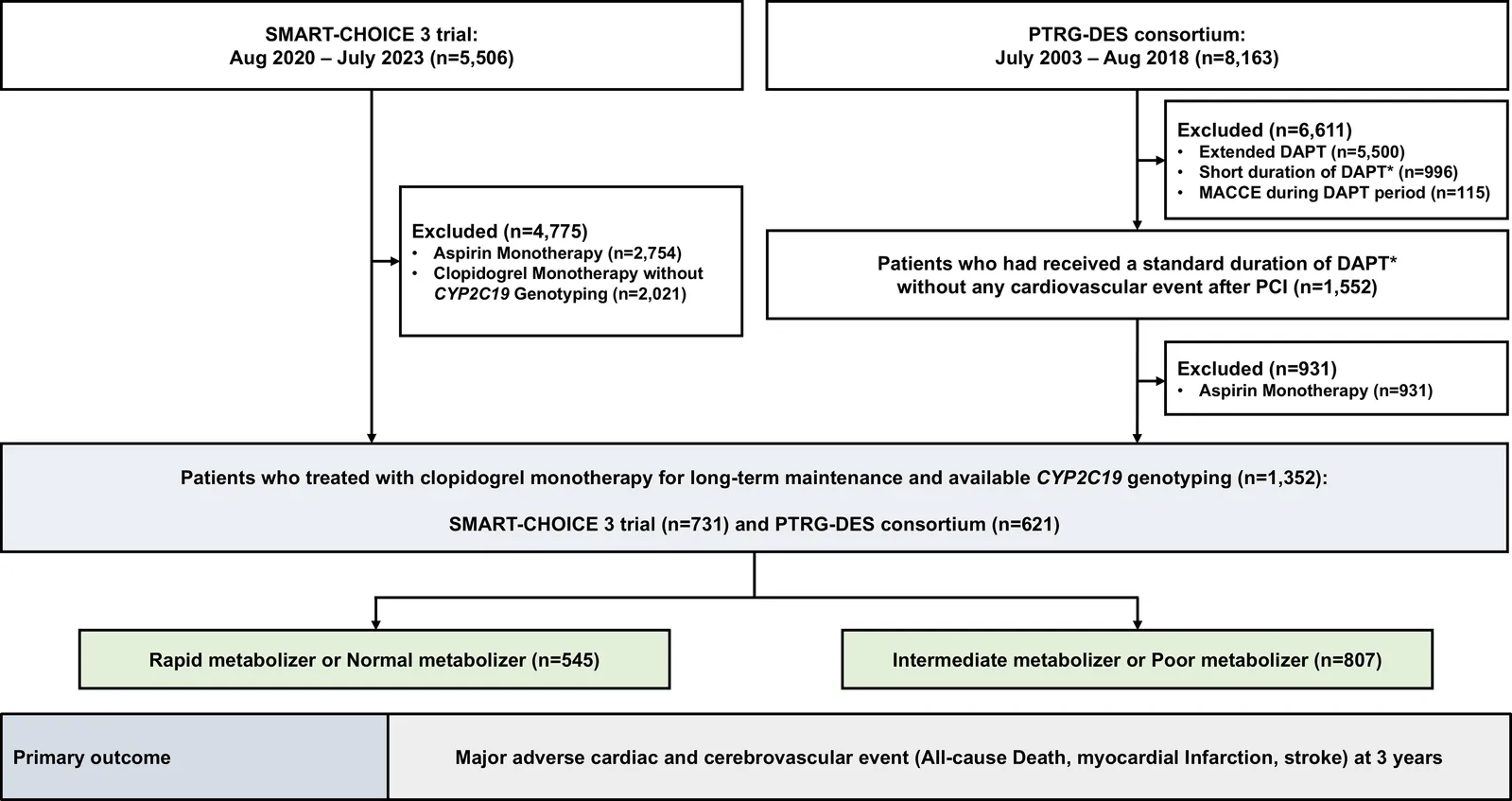
Study Flow The study population was derived from the SMART-CHOICE 3 and PTRG-DES dataset. ∗12 months for myocardial infarction; 6 months for any other events requiring PCI. CYP = cytochrome P450; DAPT = dual antiplatelet therapy; PCI = percutaneous coronary intervention; PTRG-DES = Platelet Function and genoType-Related Long-term progGosis in DES-treated Patients; SMART-CHOICE 3 = SMart Angioplasty Research Team: CHoice of Optimal Anti-Thrombotic Strategy in Patients Undergoing Implantation of Coronary Drug-Eluting Stents 3.

The study protocol was approved by the institutional review board of each participating center. Written informed consent was obtained from all patients in the SMART-CHOICE 3 trial, whereas informed consent was waived for patients in the PTRG-DES consortium by the institutional review board. The study was conducted in accordance with the Declaration of Helsinki.

### Patient management and follow-up

Patient management including revascularization strategies and the use of concomitant medications other than P2Y_12_ inhibitor was performed in accordance with contemporary clinical guidelines. For the initial DAPT regimen, the choice of P2Y_12_ inhibitor in the SMART-CHOICE 3 trial was at the operator's discretion, permitting the use of clopidogrel, ticagrelor, or prasugrel in combination with aspirin before randomization.^4^ In the PTRG-DES consortium, the DAPT regimen consisted exclusively of aspirin plus clopidogrel.^10^

All patients included in this analysis underwent *CYP2C19* genotyping using genomic DNA extracted from peripheral blood. The *CYP2C19∗2* (rs4244285), *∗3* (rs4986893), and ∗17 (rs12248560) allelic variants were analyzed via polymerase chain reaction followed by direct sequencing or single nucleotide polymorphism analysis. Genomic testing was conducted using standardized platforms, including the ABI PRISM 3730xl, the Spartan RX system, and the PSQ 96 MA pyrosequencer (Supplemental Table 1). The detailed criteria for the assignment of predicted *CYP2C19* phenotypes based on *CYP2C19* genotypes are provided in Supplemental Table 2.

Patients in the SMART-CHOICE 3 trial were followed through scheduled outpatient visits or telephone interviews at 6 and 12 months after randomization, and annually thereafter. At each follow-up, active surveillance was conducted through comprehensive history reviews, physical examinations, and assessment of adherence to the study medication. For the PTRG-DES consortium, follow-up data were retrospectively updated through review of electronic medical records.

### Study outcome

The primary outcome was major adverse cardiac and cerebrovascular events (MACCE), defined as a composite of all-cause death, myocardial infarction, and stroke at 3 years after transition to clopidogrel monotherapy. Secondary outcomes included individual components of MACCE, any revascularization, and major bleeding.

Death of unknown cause was considered cardiac death according to the definitions of the Academic Research Consortiums.^11^ Myocardial infarction was defined according to the fourth universal definition of myocardial infarction.^12^ Stroke was defined as abrupt-onset neurological sign or symptoms due to ischemia or hemorrhage within the brain, spinal cord, or retina.^13^ Major bleeding was defined as Bleeding Academic Research Consortium type 3 or 5 bleeding.^14^

### Statistical analysis

Categorical variables are presented as frequencies with percentages, and continuous variables as mean ± SD or median (IQR) according to their distribution, assessed by Kolmogorov-Smirnov test. Group comparisons were performed using the chi-square test or Fisher’s exact test for categorical variables, and Student's *t*-test or Mann-Whitney *U* test for continuous variables. Cumulative event rates were estimated using Kaplan-Meier estimates and differences were assessed with the log-rank test. The Cox proportional-hazards model was used to calculate HRs and 95% CIs for the primary and secondary outcomes including MACCE, all-cause death, myocardial infarction, stroke, any revascularization, and major bleeding. To account for potential confounders, a multivariable Cox regression model was used. The model was adjusted for clinically relevant covariates and variables with significant baseline differences, including age, sex, clinical presentation with ACS, hypertension, diabetes mellitus, chronic kidney disease, previous stroke, and complex PCI. The assumption of proportionality was assessed by Schoenfeld residuals and graphically by log-log plots. Cox proportional hazard models satisfied the proportional-hazards assumption. There were no missing data among any of the covariates included in the multivariable Cox regression models. No imputation methods for missing data were performed.

All analyses were 2-tailed, and a *P* value of <0.05 was considered statistically significant. All statistical analyses were conducted using R version 4.4.2 (R Foundation for Statistical Computing).

## Results

### Study population and baseline characteristics

Among the total of 1,352 patients (mean age, 64.4 ± 9.9 years; 873 men [75.5%]), 545 patients (40.3%) were classified as the RM/NM group (RM, n = 10 [0.7%]; NM, n = 535 [39.6%]), and 807 patients (59.7%) as the IM/PM group (IM, n = 635 [47.0%]; PM, n = 172 [12.7%]). The mean age was comparable between the groups (64.3 ± 10.0 vs 64.5 ± 9.8 years; *P* = 0.736), with no significant differences in sex distribution and clinical presentation (Table 1). Most cardiovascular risk factors were also similarly distributed, except for previous stroke, which was more frequent in the IM/PM group than in the RM/NM group (6.3% vs 3.5%; *P* = 0.029) (Table 1). Laboratory measurements and concomitant medications did not differ between the groups.

**Table 1:** Baseline Clinical Characteristics According to *CYP2C19* Genotype

	Overall (N = 1,352)	RM/NM (n = 545)	IM/PM (n = 807)	*P* Value
Demographics				
Age, y	64.4 ± 9.9	64.3 ± 10.0	64.5 ± 9.8	0.736
Male, n (%)	873 (64.6)	341 (62.6)	532 (65.9)	0.227
Body mass index, kg/m^2^	25.1 ± 3.2	25.0 ± 3.2	25.2 ± 3.2	0.280
Diagnosis at index PCI				0.547
Chronic coronary syndrome	429 (31.7)	168 (30.8)	261 (32.3)	
Unstable angina	470 (34.8)	184 (33.8)	286 (35.4)	
Non–ST-segment elevation myocardial infarction	291 (21.5)	120 (22.0)	171 (21.2)	
ST-segment elevation myocardial infarction	162 (12.0)	73 (13.4)	89 (11.0)	
Risk factors				
Hypertension	864 (63.9)	337 (61.8)	527 (65.3)	0.213
Diabetes mellitus	492 (36.4)	192 (35.2)	300 (37.2)	0.502
Dyslipidemia	1,035 (76.6)	418 (76.7)	617 (76.5)	0.970
Current smoking	174 (12.9)	75 (13.8)	99 (12.3)	0.470
Chronic kidney disease	231 (17.1)	100 (18.3)	131 (16.2)	0.347
Previous stroke	70 (5.2)	19 (3.5)	51 (6.3)	0.029
Peripheral vascular disease	27 (2.0)	9 (1.7)	18 (2.2)	0.486
High bleeding risk defined by ARC	221 (16.3)	84 (15.4)	137 (17.0)	0.492
Laboratory findings				
Hemoglobin, g/dL	13.6 ± 1.6	13.7 ± 1.6	13.6 ± 1.7	0.254
Platelet, ∗10^3^/mm^3^	221.2 ± 70.3	222.0 ± 70.7	220.7 ± 70.0	0.728
GFR (MDRD), mL/min/1.73 m^2^	80.8 ± 23.9	81.4 ± 24.5	80.3 ± 23.6	0.417
HbA_1c_, %	6.5 ± 1.1	6.6 ± 1.1	6.5 ± 1.1	0.806
Total cholesterol, mg/dL	142.3 ± 41.8	145.3 ± 42.1	140.2 ± 41.6	0.031
LDL cholesterol, mg/dL	76.1 ± 34.5	78.8 ± 34.9	74.3 ± 34.1	0.020
HDL cholesterol, mg/dL	46.6 ± 11.7	46.0 ± 11.6	47.0 ± 11.9	0.129
Triglyceride, mg/dL	124.0 ± 82.6	127.3 ± 93.9	121.8 ± 74.0	0.257
LV ejection fraction, %	59.8 ± 9.6	59.4 ± 9.7	60.0 ± 9.5	0.288
Medication				
Aspirin	0 (0)	0 (0)	0 (0)	1.000
Clopidogrel	1,352 (100.0)	545 (100.0)	807 (100.0)	1.000
Statin	1,095 (81.0)	437 (80.2)	658 (81.5)	0.581
Beta-blocker	636 (47.0)	253 (46.4)	383 (47.5)	0.749
ACE inhibitor or ARB	669 (49.5)	267 (49.0)	402 (49.8)	0.809
Proton pump inhibitor	137 (10.1)	56 (10.3)	81 (10.0)	0.960
DAPT duration, d	624 (369-1,171)	596 (370-1,158)	633 (368-1,180)	0.536

ACE = angiotensin-converting enzyme; ARB = angiotensin receptor blocker; ARC = Academic Research Consortium; *CYP* = *cytochrome P450*; DAPT = dual antiplatelet therapy; GFR = glomerular filtration rate; HbA_1c_ = glycosylated hemoglobin; HDL = high density lipoprotein; IM = intermediate metabolizer; LDL = low density lipoprotein; LV = left ventricular; MDRD = Modification of Diet in Renal Disease; NM = normal metabolizer; PCI = percutaneous coronary intervention; PM = poor metabolizer; RM = rapid metabolizer.

Angiographic and procedural characteristics of the study population are summarized in Table 2. Overall, lesion complexity and procedural characteristics did not differ significantly between the 2 groups. Specifically, the proportion of complex PCI was comparable between the RM/NM and IM/PM groups (53.6% vs 56.5%; *P* = 0.314).

**Table 2:** Angiographic and Procedural Characteristics According to *CYP2C19* Genotype

	Overall (N = 1,352)	RM/NM (n = 545)	IM/PM (n = 807)	*P* Value
Number of diseased vessels				0.890
1-vessel disease	597 (44.2)	237 (43.5)	360 (44.6)	
2-vessel disease	496 (36.7)	204 (37.4)	292 (36.2)	
3-vessel disease	259 (19.2)	104 (19.1)	155 (19.2)	
Multivessel disease	755 (55.8)	308 (56.5)	447 (55.4)	0.725
Location of diseased vessels				
Left main coronary artery	106 (7.8)	51 (9.4)	55 (6.8)	0.109
Left anterior descending artery	930 (68.8)	370 (67.9)	560 (69.4)	0.599
Left circumflex artery	566 (41.9)	228 (41.8)	338 (41.9)	>0.999
Right coronary artery	635 (47.0)	257 (47.2)	378 (46.8)	0.953
Complex PCI	748 (55.3)	292 (53.6)	456 (56.5)	0.314
PCI for 2-stenting for true bifurcation lesions	48 (3.6)	21 (3.9)	27 (3.3)	0.730
PCI for chronic total occlusion	169 (12.5)	64 (11.7)	105 (13.0)	0.543
PCI for unprotected left main coronary artery disease	98 (7.2)	47 (8.6)	51 (6.3)	0.135
PCI for long coronary lesions	536 (39.6)	208 (38.2)	328 (40.6)	0.391
Multivessel PCI	285 (21.1)	118 (21.7)	167 (20.7)	0.722
Multiple stent implantation (≥3 stents)	214 (15.8)	86 (15.8)	128 (15.9)	>0.999
Used devices				0.424
Drug-eluting stent	1,326 (98.1)	537 (98.5)	789 (97.8)	
Drug-coated balloons or others	26 (1.9)	8 (1.5)	18 (2.2)	
Total number of stents	1.7 ± 0.9	1.7 ± 0.9	1.6 ± 0.9	0.796
Stent length, mm	43.0 ± 29.0	42.7 ± 29.0	43.2 ± 29.0	0.735
Stent diameter, mm	2.9 ± 0.5	2.9 ± 0.5	2.9 ± 0.5	0.893

Abbreviations as in Table 1.

### Clinical outcomes according to *CYP2C19* genotype

The primary and secondary outcomes are described in Table 3. At 3 years, the risk of MACCE did not differ significantly between the RM/NM and IM/PM groups (2.2% vs 1.2%; adjusted HR: 0.53; 95% CI: 0.20-1.44; *P* = 0.215) (Figure 2). Similarly, the incidence of major bleeding was comparable between the 2 groups (2.0% vs 1.1%; adjusted HR: 0.40; 95% CI: 0.14-1.14; *P* = 0.086) (Figure 2). When clinical outcomes were analyzed according to the number of *CYP2C19* loss-of-function alleles, no significant trend in event rates was observed with an increasing number of loss-of-function alleles for either MACCE (RM/NM 2.2% vs IM 1.3% vs PM 1.2%; overall log-rank *P* = 0.365) or major bleeding (RM/NM 2.0% vs IM 1.2% vs PM 0.7%; overall log-rank *P* = 0.154) (Supplemental Figure 1).

**Table 3:** Clinical Outcomes According to *CYP2C19* Genotype

	RM/NM (n = 545)	IM/PM (n = 807)	Unadjusted HR (95% CI)	Adjusted HR^a^ (95% CI)	*P* Value
MACCE^b^	9 (2.2)	7 (1.2)	0.53 (0.20-1.43)	0.53 (0.20-1.44)	0.215
Secondary outcomes					
All-cause death	2 (0.4)	4 (0.6)	1.37 (0.25-7.48)	1.58 (0.29-8.69)	0.599
Myocardial infarction	3 (0.8)	1 (0.1)	0.23 (0.02-2.20)	0.23 (0.02-2.27)	0.209
Any revascularization	27 (6.1)	24 (3.8)	0.61 (0.35-1.05)	0.58 (0.33-1.01)	0.052
Stroke	4 (1.0)	2 (0.5)	0.34 (0.06-1.88)	0.31 (0.05-1.79)	0.190
Major bleeding	9 (2.0)	6 (1.1)	0.46 (0.16-1.28)	0.40 (0.14-1.14)	0.086

Data are expressed as event number (cumulative incidence by Kaplan-Meier estimates) at 3 years.

MACCE = major adverse cardiac and cerebrovascular event; other abbreviations as in Table 1.

a Adjusted for variables of age, sex, acute coronary syndrome presentation, hypertension, diabetes mellitus, chronic kidney disease, previous stroke, and complex PCI.

b MACCE was a composite of all-cause death, myocardial infarction, and stroke.

**Figure 2:**
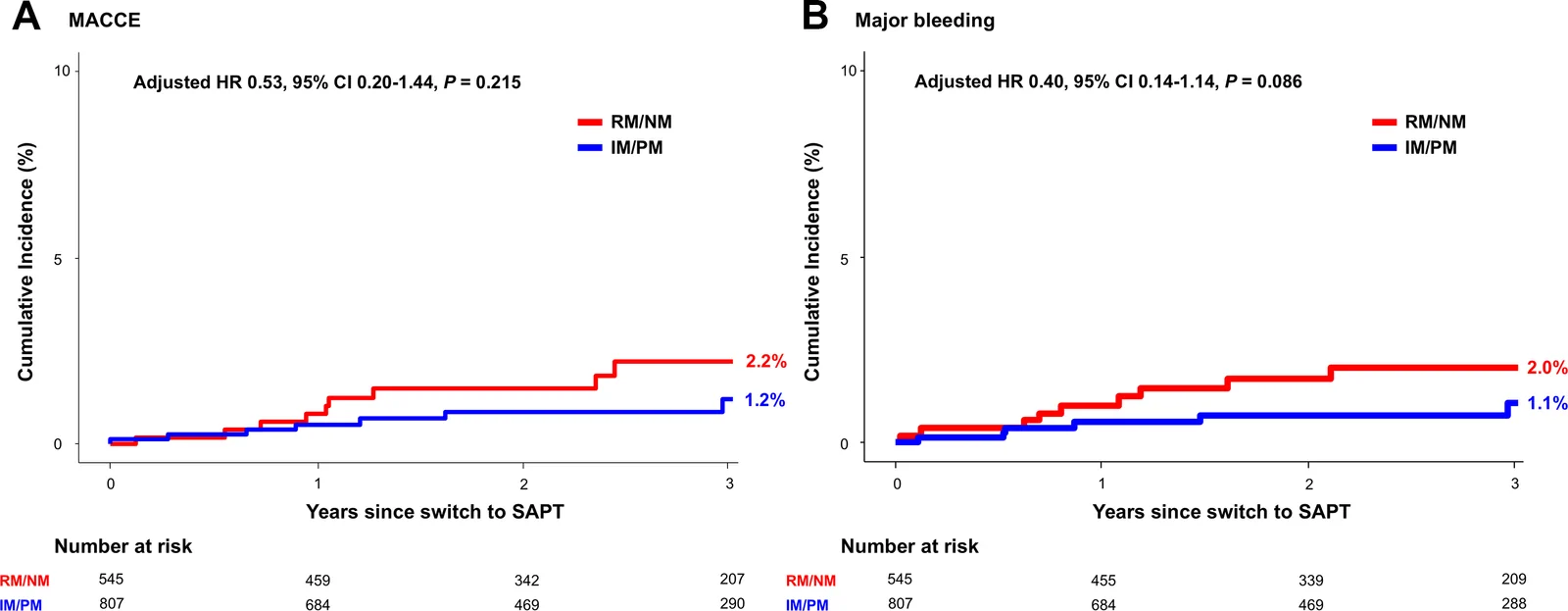
Primary Outcome and Major Safety Outcome According to *CYP2C19* Genotype Kaplan-Meier curves with cumulative incidence of (A) MACCE and (B) major bleeding according to *CYP2C19* genotype are shown. *CYP* = *cytochrome P450*; IM = intermediate metabolizer; NM = normal metabolizer; MACCE = major adverse cardiac and cerebrovascular events; PM = poor metabolizer; RM = rapid metabolizer; SAPT = single antiplatelet therapy.

Figure 3 presents the prognostic impact of *CYP2C19* genotype across the diverse subgroups. Subgroup analyses demonstrated that the association between *CYP2C19* genotype and clinical outcomes was consistent across a range of clinical settings. No significant interactions were observed in any subgroups, including those stratified by age, sex, initial clinical presentation, diabetes mellitus, high bleeding risk, proton pump inhibitor, DAPT duration, complex PCI, and study cohort.

**Figure 3:**
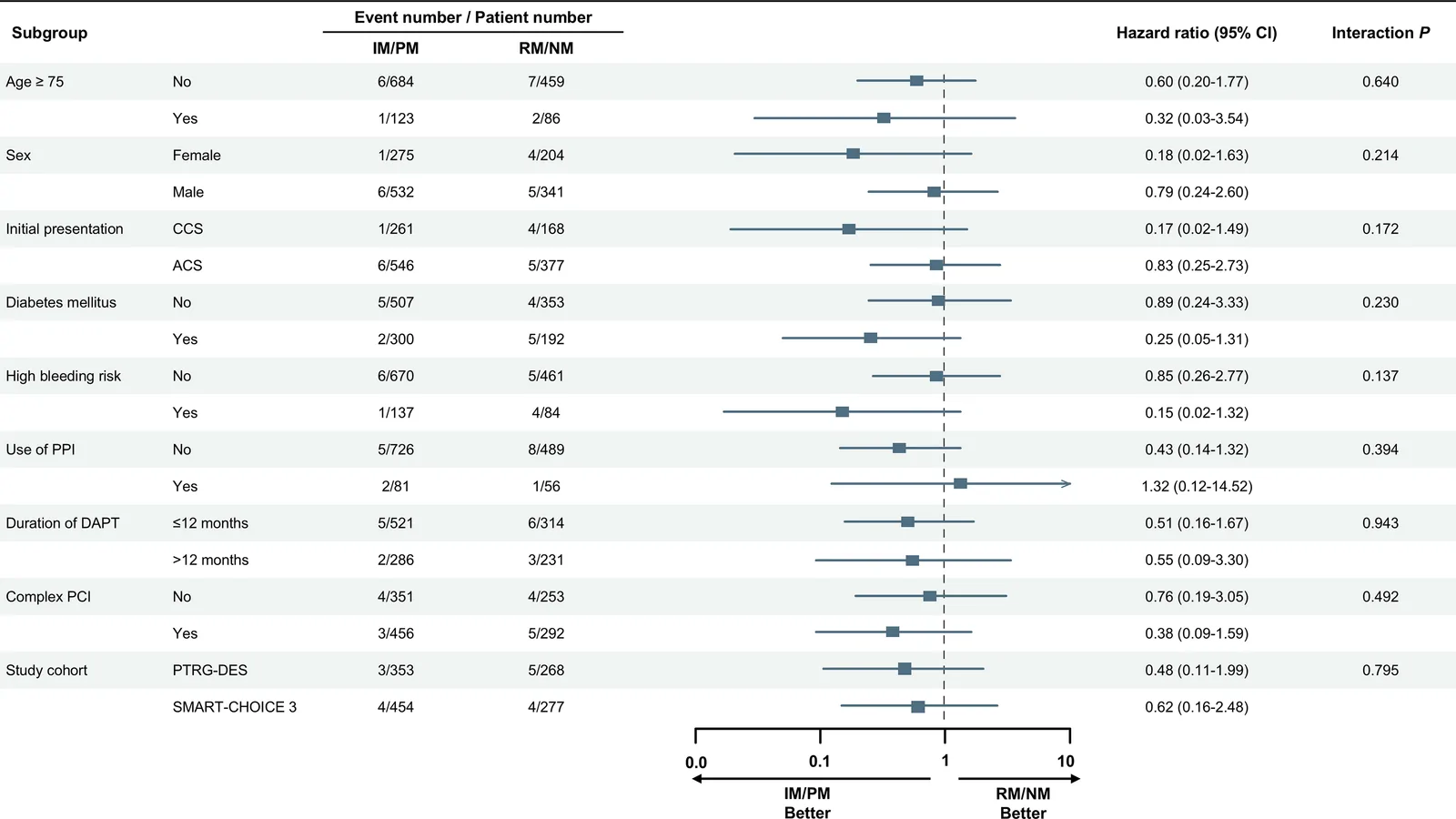
Subgroup Analysis Regarding Primary Outcomes The effects of *CYP2C19* genotype on primary outcome are shown across various subgroups. ACS = acute coronary syndrome; CCS = chronic coronary syndrome; PPI = proton pump inhibitor; other abbreviations as in Figures 1 and 2.

## Discussion

To the best of our knowledge, this study provides the first patient-level pooled evidence on the association between *CYP2C19* genotype and clinical outcomes during chronic clopidogrel monotherapy after PCI, using data from the SMART-CHOICE 3 trial and the nationwide PTRG-DES consortium. We found that the *CYP2C19* genotype was not associated with significant differences in the risks of MACCE and major bleeding among stable patients receiving clopidogrel monotherapy after completion of the standard duration of DAPT, and these findings were consistent across diverse subgroups (Central Illustration).

**Central Illustration:**
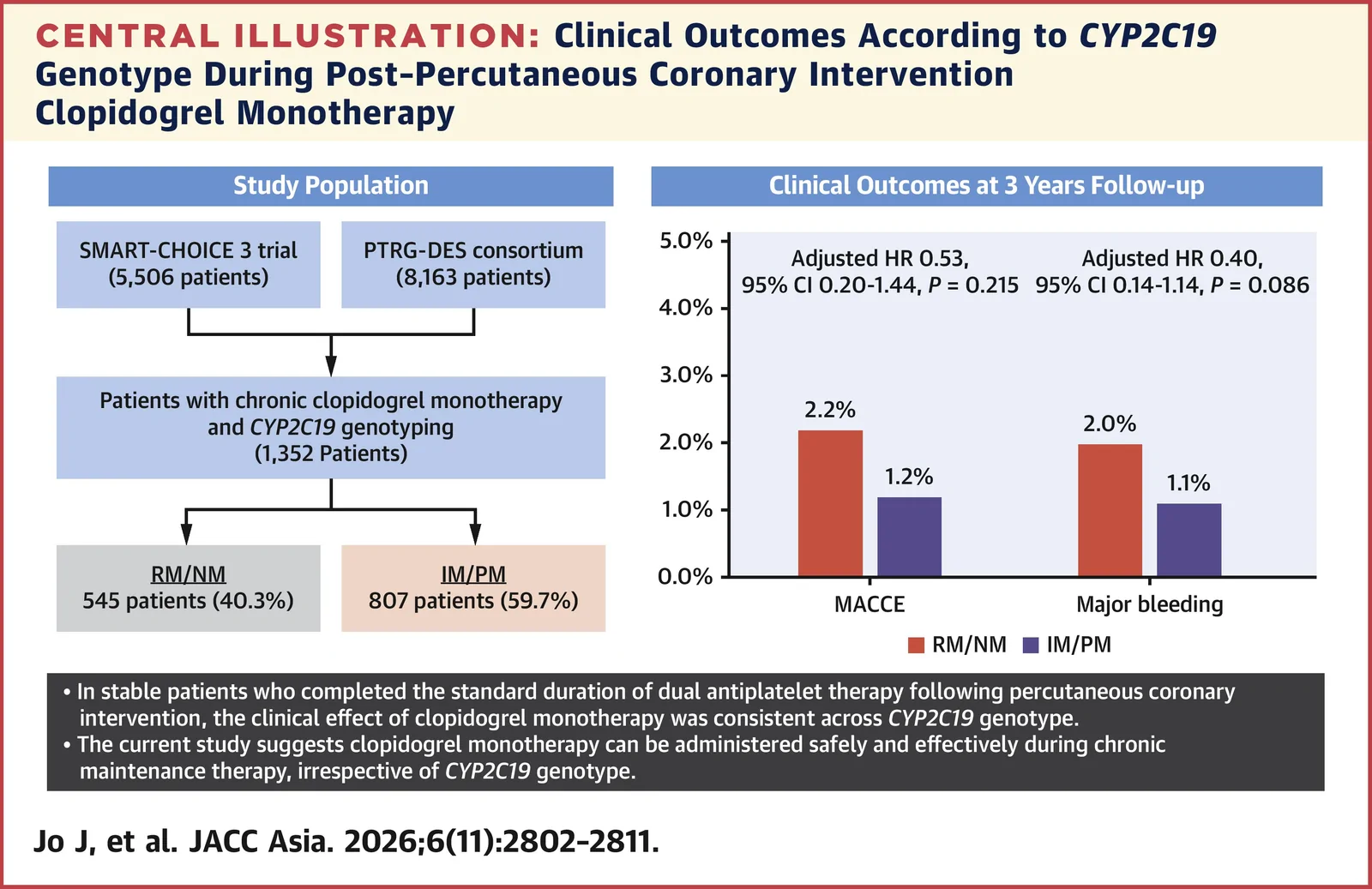
Clinical Outcomes According to *CYP2C19* Genotype During Post–Percutaneous Coronary Intervention Clopidogrel Monotherapy The current study evaluated 3-year clinical outcomes according to *CYP2C19* genotype in PCI-treated patients with chronic clopidogrel monotherapy, using data from the SMART-CHOICE 3 trial and the nationwide PTRG-DES registry. The *CYP2C19* genotype was not associated with significant differences in the risks of MACCE and major bleeding. CYP = cytochrome P450; IM = intermediate metabolizer; NM = normal metabolizer; MACCE = major adverse cardiac and cerebrovascular events; PCI = percutaneous coronary intervention; PM = poor metabolizer; PTRG-DES = Platelet Function and genoType-Related Long-term progGosis in DES-treated Patients; RM = rapid metabolizer; SMART-CHOICE 3 = SMart Angioplasty Research Team: CHoice of Optimal Anti-Thrombotic Strategy in Patients Undergoing Implantation of Coronary Drug-Eluting Stents 3.

DAPT with aspirin and a P2Y_12_ inhibitor is a cornerstone of pharmacologic management after PCI.^1^^,^^2^^,^^15^ However, although antiplatelet therapy reduces ischemic events, it inevitably increases bleeding complications. Among the multiple factors contributing to adverse outcomes after PCI, the degree of platelet inhibition achieved with antiplatelet therapy plays a key role.^16^ Over the past 2 decades, growing interest has focused on platelet function testing and genetic testing to guide the selection of oral P2Y_12_ inhibitor, with the goal of optimizing platelet inhibition in patients undergoing PCI.^17^, ^18^, ^19^ Genetic testing aims to identify *CYP2C19* variants, particularly loss-of-function alleles, associated with impaired clopidogrel-mediated antiplatelet effects.^20^ *CYP2C19* genotyping offers several advantages because genotype remains stable over time and can be assessed rapidly at the bedside without previous clopidogrel exposure, making it particularly useful in ACS when selecting a P2Y_12_ inhibitor. However, genotype does not always correspond to platelet phenotype (eg, high platelet reactivity), because platelet reactivity is also influenced by multiple nongenetic factors.^16^^,^^17^

Previous evidence has shown that carriage of *CYP2C19* loss-of-function allele is associated with reduced levels of clopidogrel's active metabolite, which correlates with high platelet reactivity phenotype and a higher risk of ischemic events such as stent thrombosis and myocardial infarction during clopidogrel-based DAPT after PCI.^9^^,^^20^ However, the impact of platelet inhibition, and of *CYP2C19* genotype, on clinical events may vary over time according to the balance of underlying thrombotic risk (eg, clinical presentation, lesion complexity, and overall clinical risk stratification) and bleeding risk.^15^ Although our previous report included DES-treated East Asian patients who are often considered to have a relatively lower thrombogenic milieu, carriage of *CYP2C19* loss-of-function alleles was associated with a 42% higher risk of ischemic events, particularly within the first year after PCI in patients presenting with ACS.^9^ Furthermore, the randomized POPular Genetics (Patient Outcome after primary PCI Genetics) trial and the study by Azzahhafi et al^18^^,^^19^ showed that *CYP2C19* genotype-guided de-escalation to clopidogrel in noncarriers reduced bleeding without increasing ischemic risk, compared with standard DAPT using ticagrelor or prasugrel in patients with ACS. These findings suggest that, during the early phase of ACS presentation, *CYP2C19* loss-of-function carriers may require more potent platelet inhibition to achieve ischemic risk reduction comparable to that seen in noncarriers treated with clopidogrel.^18^^,^^19^

The risks of ischemic and bleeding events during antiplatelet therapy are dynamic rather than fixed, necessitating ongoing reassessment of net clinical benefit. Ischemic risk is greatest in the first month after PCI and declines thereafter, whereas bleeding risk can persist as long as antiplatelet therapy is continued.^15^ Accordingly, the net benefit of antiplatelet therapy shifts over time as the balance between ischemia and bleeding risks evolves.^15^ The present study evaluated clopidogrel monotherapy as chronic maintenance in patients who completed the standard duration of DAPT following PCI. The temporal decline in thrombotic risk likely reflects progressive re-endothelialization of the stented segments and gradual stabilization of the vascular and hematologic milieu with medical therapy, which together mitigate the overall risk of recurrent ischemic events.^21^ Consequently, the clinical impact of *CYP2C19* genetic variation on ischemic outcomes may be attenuated in the stabilized chronic phase compared with early post-PCI period. In this context, the HOST-EXAM and SMART-CHOICE 3 trials have established that clopidogrel monotherapy is superior to aspirin for long-term antiplatelet maintenance following PCI in East Asian patients.^3^^,^^4^ This clinical superiority is observed despite the substantially higher prevalence of *CYP2C19* loss-of-function alleles in East Asian populations (∼65%) than in Western populations (25%-30%).^22^^,^^23^ Given that carriage of a *CYP2C19* loss-of-function allele was not associated with a higher risk of MACCE in our study, reduced clopidogrel responsiveness linked to this variant during acute-phase DAPT may not translate into clinical failure of single antiplatelet therapy during chronic maintenance therapy. Taken together, clopidogrel monotherapy appears to provide adequate protection against thrombotic events even in carriers of loss-of-function alleles, while avoiding the excess bleeding associated with more intensive platelet inhibition (eg, prolonged DAPT or monotherapy with a potent P2Y_12_ inhibitor).

### Study limitations

First, this study is subject to the inherent limitations of an observational analysis of registry data. Second, in the SMART-CHOICE 3 trial, *CYP2C19* genotyping was introduced mid-study in patients assigned to clopidogrel monotherapy. As a result, not all trial participants underwent genotyping, which may have introduced survivor bias because patients with early fatal events could have been excluded, potentially contributing to the overall low event rates. Third, given the relatively low event rates during the chronic maintenance phase and selective inclusion of patients who remained event-free during initial standard DAPT, our study was likely underpowered to detect modest differences in clinical outcomes according to *CYP2C19* genotype. A post hoc analysis based on Schoenfeld's formula indicated that 80% statistical power was achieved only for effect size of HR (≤0.24 or ≥4.16). Accordingly, the possibility of a type II error cannot be excluded, and our findings should be considered hypothesis-generating rather than definite evidence of the absence of a genotype effect. Fourth, secondary outcome and subgroup analyses were performed without formal adjustment for multiple testing. Therefore, these findings should be interpreted as exploratory. Finally, our study was conducted exclusively in an East Asian population, which may differ from Western populations in clinical characteristics, including cardiovascular risk factors and comorbidity burden, as well as in baseline ischemic and bleeding risk. However, the unique characteristics of East Asian populations, particularly the approximately 2-fold higher prevalence of *CYP2C19* loss-of-function alleles compared with Western populations, may provide more informative clinical evidence regarding the contribution of these variants to ischemic events during chronic clopidogrel monotherapy after PCI.

## Conclusions

In stable patients who completed the standard duration of DAPT following PCI, the clinical effect of clopidogrel monotherapy was consistent across *CYP2C19* genotypes. These findings provide clinical reassurance that clopidogrel monotherapy can be used safely and effectively during chronic maintenance therapy, irrespective of *CYP2C19* genotype.

## Funding Support and Author Disclosures

The SMART-CHOICE 3 trial was supported by a research grant from Dong-A ST. The PTRG-DES registry was supported by the Platelet-Thrombosis Research Group under the Korean Society of Interventional Cardiology. Dr Song has received honoraria for lectures from AstraZeneca, Daiichi-Sankyo, Sanofi, Bayer Korea, and Samjin Pharmaceutical. Dr Joo has received honoraria for lectures from AstraZeneca, Hanmi, Samjin, Dong-A, HK inno.N Pharmaceuticals, and DIO Medical Ltd. Dr Jeong has received honoraria for lectures from AstraZeneca, Daiichi-Sankyo, Sanofi, Han-mi Pharmaceuticals, and Yuhan Pharmaceuticals, as well as research grants or support from Yuhan Pharmaceuticals and U&I Corporation. Dr Hahn has received funding from the National Evidence-based Healthcare Collaborating Agency, the Ministry of Health & Welfare, Korea, Abbott Vascular, Biosensors, Biotronik, Boston Scientific, Daiichi-Sankyo, Donga-ST, Hanmi Pharmaceutical, and Medtronic Inc. All other authors have reported that they have no relationships relevant to the contents of this paper to disclose.
