# Supplementary material for: Prognostic Implication of CYP2C19 Genotype During Chronic Clopidogrel Monotherapy After Percutaneous Coronary Intervention
Source: JACC Asia. 2026 Nov 3;6(11):2802–11. doi: 10.1016/j.jacasi.2026.08.034 (PMC13750892; doi:10.1016/j.jacasi.2026.08.034)
Supplement: Supplementary file 1 [file mmc1.docx]

**SUPPLEMENTAL MATERIAL**

**Prognostic Implication of *CYP2C19* Genotype During Chronic Clopidogrel Monotherapy**

**After Percutaneous Coronary Intervention**

Jinhwan Jo, MD^a†^, Soo-Youn Lee, MD, PhD^b†^, Young-Hoon Jeong, MD, PhD^a*^, Joo-Yong Hahn, MD, PhD^b*^, et al.

**Table of Contents**

- **Supplemental Figure 1**
- **Supplemental Tables 1-2**

**Supplemental Figure 1. Primary Outcome and Major Safety Outcome According to Number of *CYP2C19* Loss-of-function Allele**

Kaplan-Meier curves with cumulative incidence of (A) MACCE and (B) Major bleeding according to *CYP2C19* Loss-of-function Allele are shown.

Abbreviations: CI, confidence interval; *CYP*, *cytochrome P450*; DAPT, dual antiplatelet therapy; HR, hazard ratio; IM, intermediate metabolizer; NM, normal metabolizer; MACCE, major adverse cardiac and cerebrovascular events; PM, poor metabolizer; RM, rapid metabolizer; SAPT, single antiplatelet therapy.

**Supplemental Table 1. Method of Genotyping and Number of Patients in Each Registry**

| **Registry** | **Name** | **Method** | **Number** |
| --- | --- | --- | --- |
| **SMART-CHOICE 3^1^**  **(n=731)** | SMC | ABI PRISM® 3100 genetic analyzer (Applied Biosystems, Foster City, USA) | 731 |
| **PTRG-DES**  **(n=621)** | GENIUS^2^ | PSQ 96MA Pyrosequencer (Pyrosequencing AB, Uppsala, Sweden) | 59 |
|  | Catholic^3^ | ABI PRISM® 3100 genetic analyzer (Applied Biosystems, Foster City, USA) | 93 |
|  | GNUH^4^ | ABI PRISM® 3100 genetic analyzer (Applied Biosystems, Foster City, USA) | 29 |
|  | SMC^5^ | ABI PRISM® 3100 genetic analyzer (Applied Biosystems, Foster City, USA) | 28 |
|  | WSC^6^ | ABI PRISM® 3100 genetic analyzer (Applied Biosystems, Foster City, USA) | 14 |
|  | Dong-A^7^ | Spartan RX™ system (Spartan Bioscience, Ottawa, Canada) | 390 |
|  | CNU^8^ | ABI PRISM® 3100 genetic analyzer (Applied Biosystems, Foster City, USA) | 8 |

Abbreviations: PTRG-DES, Platelet Function and genoType-Related Long-term progGosis in DES-treated Patients; SMART-CHOICE 3, SMart Angioplasty Research Team: CHoice of Optimal Anti-Thrombotic Strategy in Patients Undergoing Implantation of Coronary Drug-Eluting Stents 3.

**Supplemental Table 2. Assignment of Predicted *CYP2C19* Phenotype Based on Genotyping**

| **Predicted *CYP2C19* Phenotype** | | **Genotype** | | **Examples of *CYP2C19* Genotype** | |
| --- | --- | --- | --- | --- | --- |
| Rapid Metabolizer | An individual carrying 1 normal function allele and 1 increased function allele | | *1/*17 | |  |
| Normal Metabolizer | An individual carrying 2 normal function alleles | | *1/*1 | |  |
| Intermediate Metabolizer | An individual carrying 1 normal function allele and 1 no function allele or 1 increased function allele and 1 no function allele | | *1/*2 | |  |
|  |  |  | *1/*3 | |  |
|  |  |  | *2/*17 | |  |
|  |  |  | *3/*17 | |  |
| Poor Metabolizer | An individual carrying 2 no function alleles | | *2/*2 | |  |
|  |  |  | *2/*3 | |  |
|  |  |  | *3/*3 | |  |

Abbreviations: *CYP,* *cytochrome P450*.

**REFERENCES**

**1.** Choi KH, Park YH, Lee JY, et al. Efficacy and safety of clopidogrel versus aspirin monotherapy in patients at high risk of subsequent cardiovascular event after percutaneous coronary intervention (SMART-CHOICE 3): a randomised, open-label, multicentre trial. *Lancet*. 2025;405:1252-1263.

**2.** Joo HJ, Ahn SG, Park JH, et al. Effects of genetic variants on platelet reactivity and one-year clinical outcomes after percutaneous coronary intervention: A prospective multicentre registry study. *Sci Rep.* 2018;8:1229.

**3.** Kim HS, Chang K, Koh YS, et al. CYP2C19 poor metabolizer is associated with clinical outcome of clopidogrel therapy in acute myocardial infarction but not stable angina. *Circ Cardiovasc Genet.* 2013;6:514-21.

**4.** Jeong YH, Kim IS, Park Y, et al. Carriage of cytochrome 2C19 polymorphism is associated with risk of high post-treatment platelet reactivity on high maintenance-dose clopidogrel of 150 mg/day: results of the ACCEL-DOUBLE (Accelerated Platelet Inhibition by a Double Dose of Clopidogrel According to Gene Polymorphism) study. *JACC Cardiovasc Interv.* 2010;3:731-41.

**5**. Lee GY, Hahn JY, Lee SY, et al. Adjunctive cilostazol versus high maintenance dose of clopidogrel in patients with hyporesponsiveness to chronic clopidogrel therapy. *Yonsei Med J*. 2013;54:34-40.

**6**. Ahn SG, Lee SH, Yoon JH, et al. Different prognostic significance of high on-treatment platelet reactivity as assessed by the VerifyNow P2Y12 assay after coronary stenting in patients with and without acute myocardial infarction. *JACC Cardiovasc Interv*. 2012;5:259-267.

**7.** Zhang HZ, Kim MH, Han JY, Jeong YH. Defining predictive values using three different platelet function tests for CYP2C19 phenotype status on maintenance dual antiplatelet therapy after PCI. *Platelets*. 2014;25:285-291.

**8.** Lee SY, Lee JH, Lee SM, Bae JW, Cho MC. Clinical Impact of Platelet Reactivity and Gene Polymorphisms Tests in Patients with Interventional Procedures for Coronary Artery Disease. *J Am Coll Cardiol*. 2015;65:S10-S11.
